# Comparison of Adult and Adolescent Preferences Toward EQ-5D-Y-3L Health States

**DOI:** 10.1016/j.jval.2021.03.019

**Published:** 2021-09

**Authors:** Valentina Prevolnik Rupel, Juan M. Ramos-Goñi, Marko Ogorevc, Simone Kreimeier, Kristina Ludwig, Wolfgang Greiner

**Affiliations:** 1 Institute for Economic Research, Ljubljana, Slovenia; 2 Maths in Health B.V., Rotterdam, The Netherlands; 3 Department of Health Economics and Health Care Management, School of Public Health, Bielefeld University, Bielefeld, Germany

**Keywords:** adult preferences, children and adolescents’ preferences, discrete choice experiment, EQ-5D-Y-3L, health-related quality of life, valuation

## Abstract

**Objectives:**

The international EQ-5D-Y-3L valuation protocol suggests obtaining preferences for EQ-5D-Y-3L health states from a sample of the general adult population. There is discussion around involving children and adolescents in the processes of preference elicitation and decision making. The objective of this study was to compare the preferences for EQ-5D-Y-3L obtained from adolescents for themselves to those of adults considering a hypothetical 10-year-old child in Germany, Slovenia, and Spain.

**Methods:**

A sample of a minimum of 700 adolescents and 1000 adults in each country was recruited through online panels. An online discrete choice experiment was used to obtain health-state preferences. For the purposes of comparison, all coefficients were rescaled to a 1 (best) to 0 (worst) scale. The differences between preferences in both samples were analyzed via the relative attribute importance of health dimensions from the mixed logit models.

**Results:**

Statistically significant differences between the preferences for EQ-5D-Y-3L states given by the 2 samples were observed in all 3 countries. The overall relative attribute importance was similar between adolescents and adults; adolescents usually gave more importance to mobility and self-care, and less to anxiety/depression. The rank-order of the dimension levels between adults and adolescents differs in all 3 countries.

**Conclusions:**

Preferences toward EQ-5D-Y-3L states differ if estimated by adults taking the perspective of a child or by the adolescents themselves. Although it seems possible to obtain adolescents’ preferences for inclusion in EQ-5D-Y-3L value sets, the desirability and acceptance of their preferences by researchers and decision makers need to be explored further.

## Introduction

In the past decade, there has been a general discussion about who should value health states of children and adolescents. In typical health state valuation studies, the preferences of adults from the general population are obtained as a standard approach.^1^^,^^2^ This is consistent with a public policy perspective on decision making, using the views of those members of the general population who are eligible to vote and who contribute toward financing the healthcare system through taxes or contributions.^3^ Previous research^4^ confirmed that values for EQ-5D-Y-3L (youth) health states are different compared with values for EQ-5D-3L health states; both valued by adults, but using different perspectives. This evidence suggests that EQ-5D-3L value sets should not be used to assign values to EQ-5D-Y-3L health states. The first version of the international EQ-5D-Y-3L valuation protocol was recently published, which suggests obtaining preferences toward EQ-5D-Y-3L health states from a sample of the general adult population taking the perspective of a 10-year-old child, to keep the taxpayer perspective.^5^ From the viewpoint of decision making, such an approach seems justified, because children and adolescents are generally not involved in the financing and decision making of the healthcare system.^6^ Furthermore, they are often inexperienced in making medical decisions for themselves because decisions are ultimately made by the person responsible for caring for them.^3^ At the same time, it can be argued^3^^,^^7^ that it is the child or adolescent who experiences the health states and lives with the health impairments^8^—their preferences are then directly relevant.

If we consider the involvement of children and adolescents in the preference elicitation process, their ability to value health states is questioned. There are still concerns about whether directly elicited adolescent preferences are valid and from which age children can reliably complete valuation tasks.^3^ Concerns relate to cognitive demand, linguistic limitations, comprehensive ability, and the ethical sensitivity of valuation tasks.^9^ Juniper^10^ established that children younger than 7 years had difficulties assessing utilities on a visual analog scale (VAS), whereas children aged 11 years and younger had problems understanding standard gambling exercises. Nevertheless, it has been established that children and adolescents aged 10 to 17 years could complete best-worst scaling tasks, because no inconsistencies in the worst choices were identified in adolescents compared with adults.^11^^,^^12^ Adolescents between 14 and 17 years of age can undertake pairwise comparison tasks.^13^ Thus, it may be possible to ask children and adolescents themselves about their health-state preferences, which would lead to even more discussion on the inclusion of children and adolescents’ preferences in the generation of value sets. As previously determined,^14^ discrete choice experiment (DCE) is suitable and feasible for use in valuing child health states.

Not many studies have investigated the differences between adult and adolescent preferences in the context of EQ-5D-Y-3L health states. Mott et al^14^ used a DCE and found that adult and adolescent preferences differed slightly. In the study, it appeared that adolescents were more concerned with mobility issues than the adult sample. The most important attribute levels were a lot of pain or discomfort and a lot of anxiety or depression, and the least important were some problems with usual activities, mobility, and self-care for both groups. In contrast, Dalziel et al^11^ found more significant differences in an Australian sample than in a Spanish sample when preferences were obtained by best-worst scaling. The Australian adults, answering from both perspectives (their own and that of a 10-year-old child), placed less weight on the anxiety or depression dimension and more weight on pain or discomfort than the Australian adolescents. Spanish adults and adolescents both chose pain or discomfort as the most important dimension.

The objective of this study was to explore further the differences between preferences toward EQ-5D-Y-3L health states obtained from children and adolescents themselves and from the general adult population (valuing preferences for a 10-year-old child), using DCE in 3 countries, namely, Germany, Slovenia, and Spain.

## Methods

### Sampling and Recruitment

A sample of 1700 participants (700 adolescents and 1000 adults) in each of the 3 countries, Germany, Spain, and Slovenia, was obtained through market research companies owning online panels. The adolescents’ ages were between 11 and 17 years, whereas the adults were 18 years and older. Legally, the definition of a child and an adolescent is similar in all 3 countries: a person is regarded as a child up to 18 years in Slovenia and Spain and up to 14 years in Germany; in Germany, adolescents are defined as young people aged from 14 to 18 years. The size of the adult sample was determined by the sample size recommendations of the EQ-5D-Y-3L valuation protocol.^5^ The minimum sample size for the adolescents was calculated to ensure approximately 50 observations per DCE pair (10 blocks mean 500 participants) but was increased to 70 observations per pair to allow future subgroup analysis.^15^ The quota samples were formed as follows: the Slovenian quotas were formed according to age groups (adults, 18-29, 30-39, 40-49, 50-59, 60-69, 70 years and older; adolescents, 11-14, 15-17 years), gender (female or male), and Nomenclature of Territorial Units for Statistics 2 regions (West Slovenia, East Slovenia). The German quotas were formed according to age groups (adults, 18-24, 25-29, 30-39, 40-49, 50-59, 60-69, 70 years and above; adolescents, 11-12, 13-14, 15-16, 17 years), gender (female or male), and federal states (16 states); the German samples were further controlled for education. The Spanish quotas were formed according to age groups (adults, 18-24, 25-29, 30-39, 40-49, 50-59, 60-69, 70 years and older; adolescents, 11-14, 15-17 years), gender (female or male), Nomenclature of Territorial Units for Statistics 2 regions, and education.

### EQ-5D-Y-3L

The EQ-5D-Y-3L is a patient-reported outcome measure that was designed by the EuroQol Group to measure the health-related quality of life (HRQoL) of children and adolescents aged 8 to 15 years. The standard 3-level version (3L) of the EQ-5D-Y was used in the study. The EQ-5D-Y-3L uses adapted wording in its descriptive system compared with the adult version EQ-5D-3L, which makes it easier for young people to understand. It contains 5 dimensions (youth version in brackets): mobility (MO, “mobility [walking about]”), self-care (SC, “looking after myself”), usual activities (UA, “doing usual activities”), pain or discomfort (PD, “having pain or discomfort”), and anxiety or depression (AD, “feeling worried, sad or unhappy”), with 3 levels of severity (level 1 describing no problems, level 2 describing some problems, and level 3 measuring a lot of problems) in each dimension.^16^ In the interest of brevity, the traditional dimension labels used in the adult questionnaire and their acronyms (see above) are used throughout this article. For the same reason, in dimensions PD and AD, we keep the level description “no problems,” “some problems,” and “a lot of problems,” instead of the EQ-5D-Y-3L wording of “no pain or discomfort” and “not worried, sad, or unhappy.” The EQ-5D-Y-3L has shown its feasibility in different disease areas^15^^,^^17^^,^^18^ and is being increasingly used.^19^ Up to now, the EQ-5D-Y-3L has lacked its value sets, which would enable calculation of the responses into health state utilities and the estimation of quality-adjusted life-years. Hence, it is essential to conduct preference elicitation studies to generate value sets for the EQ-5D-Y-3L instrument.

### Preparation of the Online Survey and DCE Design

In the online survey, programmed using LimeSurvey, a DCE was used to obtain EQ-5D-Y-3L health-state preferences from adults from the general population and from adolescents. Within the DCE task, 2 health states were presented next to each other, and the respondent was asked to choose the preferred health state (forced choice). The health states were described by the EQ-5D-Y-3L (Appendix 1 in Supplemental Materials found at https://doi.org/10.1016/j.jval.2021.03.019).

The experimental design followed the international valuation protocol for the EQ-5D-Y-3L.^5^ Each respondent completed 15 tasks, and the design was divided into 10 blocks yielding a total of 150 pairs. In addition, as a quality check, all the respondents completed 3 further “fixed dominant pairs,” in which one health state was considered to dominate the other logically (21123/22233, 21212/31223, 31212/32332). Overall, each respondent answered 18 DCE tasks. Besides these, the survey included additional questions, such as sociodemographic questions, questions about experience with illness, and questions about the type of tasks or questions.

The adolescents in our study valued the health states for themselves, whereas the adults valued the health states for a hypothetical 10-year-old child, as recommended by the valuation protocol^5^ and used in earlier studies.^11^^,^^20^, ^21^, ^22^

Ethical approvals for the study were obtained in all 3 countries (Slovenia, Committee of the Republic of Slovenia for Medical Ethics, No. 0120-154/2018/8 and Annex No. 0120-154/2018/15; Germany, Ethics Committee of the Bielefeld University, No. EUB 2018-172; and Spain, Comité Ético de Investigación Clínica, Hospital Nuestra Señora del Prado, No. Dictamen 32/19).

### Quality Control

Because an online survey was used, a combination of approaches to identify respondents whose choices suggested a poor level of attentiveness, engagement, or understanding was applied. The responses were quality control checked through 2 components:

1. The minimum amount of time for all the DCE tasks was set at 150 seconds. Although at the beginning the minimum amount of time was set at 300 seconds, it was established in the testing phase that nearly all the respondents failed the task—the time required was 170 to 280 seconds, which is why the minimum amount of time criterion was set at 150 seconds. Although 150 seconds may seem insufficient, overlap and bolding of the differences between the health states helped the participants and facilitated the task. The time per individual task was not considered as a validity measure.
2. Three fixed dominant pairs in which one health state logically dominated the other by definition were included, and participants were excluded if they responded incorrectly to at least 2 of the 3 pairs. A failure to see the dominance in most of the dominant pairs could indicate that the respondent was not attentive enough, did not read the description, or did not care about or understand the task.

### Data Analysis

Descriptive analyses were used to explore sample characteristics. To compare adult and adolescent preferences, mixed logit models were estimated, which took into account preference heterogeneity by the estimation of parameters for each respondent. It has been shown that mixed logit models are associated with better fit than multinomial and other models^14^^,^^23^ and, moreover, that any discrete choice model derived from random utility maximization can be approximated to any degree of accuracy by a mixed logit with appropriate choice of variables and mixing distribution.^24^

Observed heterogeneity (deterministic differences between adults and adolescents) was accommodated in the random parameters by including individual-specific covariates. When assuming that the parameters have a multivariate normal distribution, ${\text{β}}_{\text{i}}∼\text{MNV}\left(\text{β},\text{ Σ}\right)$, the vector ${\text{β}}_{\text{i}}$ can be rewritten as:

$${\text{β}}_{\text{i}}=\text{β}+\text{Π}{\text{z}}_{\text{i}}+\text{L}{\text{η}}_{\text{i}}$$

where *z*_*i*_ is a dummy variable, indicating if the person *i* is adolescent, and Π is an interaction term of differences between adolescents and adults, ${\text{η}}_{\text{i}}∼\text{N}\left(0,\text{I}\right)$, and L is the lower-triangular Cholesky factor of Σ such that ${\text{LL}}^{\text{T}}=\text{ VAR}\left({\text{β}}_{\text{i}}\right)=\text{ Σ}$.

Both samples, adult and adolescent, were pooled for each country, and a linear additive utility function was estimated with all variables dummy coded “No problems” used as base level. The null hypothesis that all differences between adults and adolescents, Π, equal 0 was tested using the Wald test and likelihood ratio test. The 2 tests involved the comparison of models with and without differences between adults and adolescents.

All coefficients were treated as random following a normal distribution and were allowed to be correlated. Because each respondent provided multiple observations, a mixed logit model was estimated using panel techniques with 1500 Halton draws from the mixing distribution.

The applied DCE technique produces the relative importance of preferences on a latent scale, meaning that this technique does not automatically provide values on the same scale. To address scaling, all the coefficients were subjected to profile-based normalization—which presents attribute importance as a proportion of the total utility induced by exchanging treatment profiles.^25^ To compare the preferences of adults and adolescents, 2 approaches were used: (1) an examination of the relative attribute importance of health dimensions and (2) an examination of implied rankings. Data analysis was done using R (4.01).

## Results

**Figure 1:**
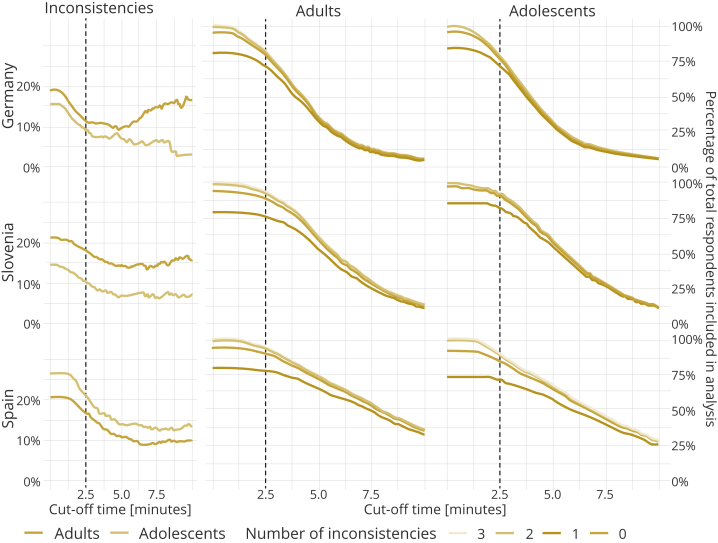
The validity of responses in adult and adolescent samples and the effect of quality criteria on the sample size by country.

Of the 3926 adult respondents and 2397 adolescent respondents who completed the survey and gave consent for their answers to be used in the analysis, a total of 3109 adults (79.2%) and 2129 adolescents (88.8%) completed the survey with an acceptable quality of responses, that is, no more than one logical inconsistency (Germany, 1030 adults and 710 adolescents; Slovenia, 1074 adults and 717 adolescents; Spain, 1005 adults and 702 adolescents). The adolescents completed the DCE quicker than the adults, with a median time of 294 seconds, whereas the median time of the adults was 313 seconds. In addition, the adolescents made fewer inconsistent decisions with the dominant pairs. At the cutoff time of 150 seconds, 85.8% of adolescents and 84.4% of adults had completed the DCE task investing enough time without logical inconsistencies. Nevertheless, differences in the validity of responses were not consistent across all 3 countries. Adolescents in Germany and Slovenia showed fewer logical inconsistencies than adults, whereas adolescents in Spain showed more, regardless of the cutoff time chosen for quality control. The validity of the responses in the adult and adolescent samples and the effect of different quality criteria on the sample size by country are shown in Figure 1. The 2 rightmost columns (adults and adolescents) present a sensitivity analysis on the cutoff for timing and number of inconsistencies. The characteristics of the adult and adolescent samples included in the analysis are shown in Tables 1 and 2, respectively. The main difference observed in the sample characteristics is the prevalence of health problems reported for the EQ-5D-Y-3L dimensions. Adults in the 3 countries reported more problems than adolescents in all dimensions. They indicated, on average, a lower score (worse health) on the EQ VAS. The median reported VAS ranges between 80 and 82 for the adults and between 90 and 91 for the adolescents, whereas the mean VAS ranges between 73.7 and 79.9 for the adults and between 86.1 and 88.5 for the adolescents.

**Table 1:** Adult sample characteristics by country.

Variables	Germany	Slovenia	Spain
Sample size			
Respondents	1030	1074	1005
Sex, n (%)			
Female	546 (53.0)	529 (49.3)	509 (50.6)
Male	482 (46.8)	545 (50.7)	496 (49.4)
Other	2 (0.2)	0 (0.0)	0 (0.0)
Age, n (%)			
18-29	165 (16.0)	154 (14.3)	125 (12.4)
30-39	153 (14.8)	172 (16.0)	182 (18.1)
40-49	152 (14.8)	194 (18.1)	286 (28.5)
50-59	201 (19.5)	194 (18.1)	236 (23.5)
60-69	149 (14.5)	183 (17.0)	136 (13.5)
≥70	210 (20.4)	177 (16.5)	40 (4.0)
MO, n (%)			
No problems	733 (71.2)	818 (76.2)	872 (86.8)
Some problems	249 (24.2)	230 (21.4)	124 (12.3)
A lot of problems	48 (4.7)	26 (2.4)	9 (0.9)
SC, n (%)			
No problems	959 (93.1)	1003 (93.4)	958 (95.3)
Some problems	67 (6.5)	66 (6.2)	46 (4.6)
A lot of problems	4 (0.4)	5 (0.5)	1 (0.1)
UA, n (%)			
No problems	795 (77.2)	873 (81.3)	859 (85.5)
Some problems	212 (20.6)	189 (17.6)	126 (12.5)
A lot of problems	23 (2.2)	12 (1.1)	20 (2.0)
PD, n (%)			
No problems	440 (42.7)	627 (58.4)	695 (69.2)
Some problems	553 (53.7)	415 (38.6)	293 (29.1)
A lot of problems	37 (3.6)	32 (3.0)	17 (1.7)
AD, n (%)			
No problems	542 (52.6)	583 (54.3)	429 (42.7)
Some problems	429 (41.6)	456 (42.5)	507 (50.4)
A lot of problems	59 (5.7)	35 (3.3)	69 (6.9)
VAS			
Mean	73.7	79.9	77.1
Standard deviation	18.6	15.7	17
Minimum	1	0	0
Maximum	100	100	100
Median	80	82	80

*Note*. Adults filled out the EQ-5D-Y-3L.

AD indicates anxiety or depression; MO, mobility; PD, pain or discomfort; SC, self-care; UA, usual activities; VAS, visual analog scale.

**Table 2:** Adolescent sample characteristics by country.

Variables	Germany	Slovenia	Spain
Sample size			
Respondents	710	717	702
Sex, n (%)			
Female	343 (48.3)	348 (48.5)	320 (45.6)
Male	367 (51.7)	369(51.5)	382 (54.4)
Other	0 (0.0)	0 (0.0)	0 (0.0)
Age, n (%)			
11-12	204 (28.7)	191 (26.6)	198 (28.2)
13-14	219 (30.9)	221 (30.8)	181 (25.8)
15-17	287 (40.4)	305 (42.5)	323 (46.0)
MO, n (%)			
No problems	646 (91.9)	657 (91.6)	665 (94.7)
Some problems	54 (7.6)	58 (8.1)	30 (4.3)
A lot of problems	10 (1.4)	2 (0.3)	7 (1.0)
SC, n (%)			
No problems	678 (95.5)	676 (94.3)	685 (97.6)
Some problems	28 (3.9)	38 (5.3)	15 (2.1)
A lot of problems	4 (0.6)	3 (0.4)	2 (0.3)
UA, n (%)			
No problems	651 (91.7)	572 (79.8)	596 (84.9)
Some problems	55 (7.8)	140 (19.5)	71 (10.1)
A lot of problems	4 (0.6)	5 (0.7)	35 (5.0)
PD, n (%)			
No problems	537 (75.6)	492 (68.6)	608 (86.6)
Some problems	167 (23.5)	218 (0.304)	92 (13.1)
A lot of problems	6 (0.8)	7 (1.0)	2 (0.3)
AD, n (%)			
No problems	477 (67.2)	398 (55.5)	357 (50.8)
Some problems	218 (30.7)	297 (41.4)	311 (44.3)
A lot of problems	15 (2.1)	22 (3.1)	34 (4.8)
VAS			
Mean	86.1	88.5	88.2
Standard deviation	13.4	14	13.6
Minimum	10	20	9
Maximum	100	100	100
Median	90	90	91

AD indicates anxiety or depression; MO, mobility; PD, pain or discomfort; SC, self-care; UA, usual activities; VAS, visual analog scale.

**Figure 2:**
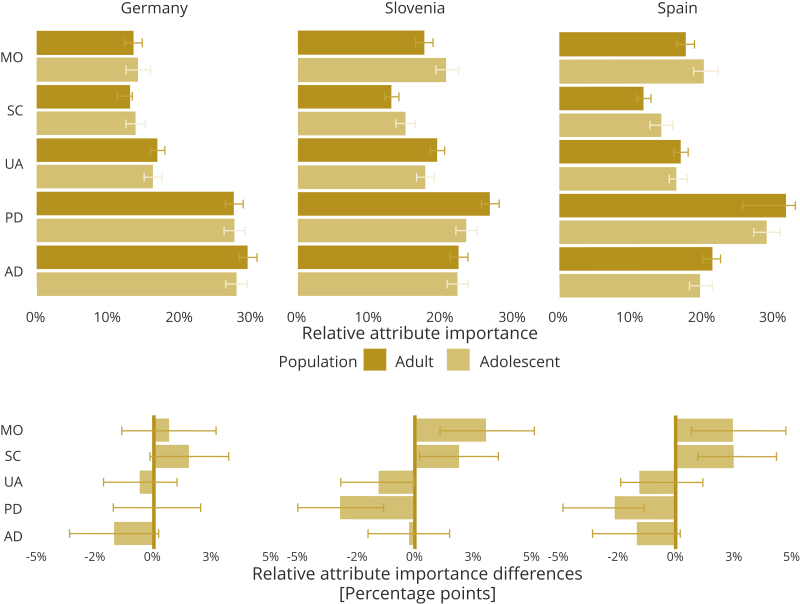
Relative importance of health dimensions and differences between adults and adolescents. *Note.* 90% confidence intervals are shown for each dimension (bootstrapped).

**Figure 3:**
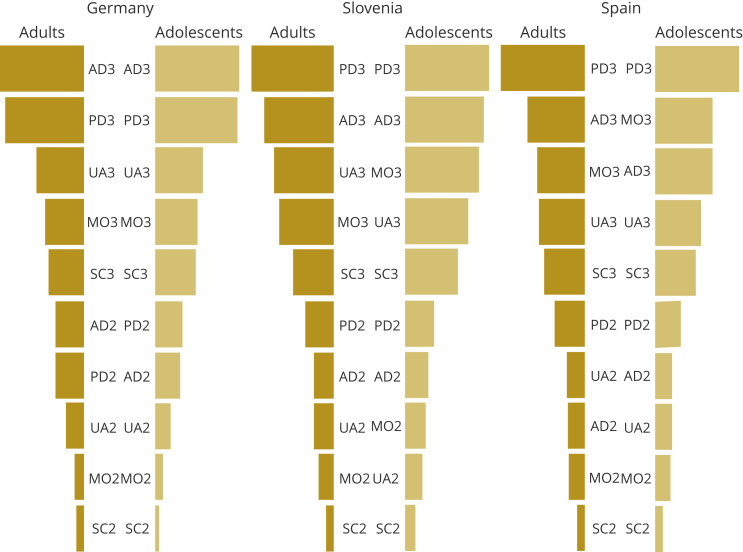
Implied rankings from the mixed logit models. *Note.* 2, some problems; 3, a lot of problems. Size of bars indicates relative importance.

All the estimated coefficients of the mixed logit models for adults were consistent and statistically significant at the 1% level, as shown in Table 3. Both the Wald test and the likelihood ratio test are statistically significant at the 1% level for all models, which means that the adolescent group differs from the adults. Specific differences in coefficients between the adult and adolescent preferences were found in all countries, although differences in Germany were significant only at the 10% level. In Germany, the adolescents indicated the SC level 3 to have a relatively stronger impact and the SC level 2 a relatively lesser impact on utility than adults. In Slovenia, the adolescents found the MO level 3 and the SC level 3 to have a stronger impact on utility than adults. In Spain, the adolescents found the AD, the UA level 3, and the PD at both levels to have a lesser impact on utility than adults. Nevertheless, particularly in Spain, differences may be attributed to differences in scale, because all the adolescent differences were positive, except SC level 3. Scale heterogeneity is a form of correlation among utility coefficients, by which the coefficients of all the included variables are larger in magnitude for some people than others. Thus, importance measures were normalized to compare attribute importance across subgroups within a sample. In Slovenia and Spain, the highest importance was given to the PD dimension, whereas in Germany only the adolescents considered PD to be the most important. The least important dimension was SC in all samples of the 3 countries (Figs. 2 and 3). Differences in relative attribute importance between the adolescents and the adults were insignificant in Germany, and in AD in all 3 countries. The overall relative attribute importance is to a large degree similar between adolescents and adults; the adolescents, however, considered MO and SC as more important and PD as less important than adults in Slovenia and Spain (Fig. 2).

**Table 3:** Results of mixed logit models for adult and adolescent preferences in the 3 countries.

Dimension	Adult coefficients	Germany			Slovenia			Spain	
		Adolescent differences	Standard deviation	Adult coefficients	Adolescent differences	Standard deviation	Adult coefficients	Adolescent differences	Standard deviation
MO2	–0.430∗	0.093	0.158	-0.576∗	-0.135	0.860∗	-0.713∗	0.176	0.306†
	(0.076)	(0.118)	(0.144)	(0.078)	(0.112)	(0.106)	(0.074)	(0.107)	(0.114)
MO3	–1.724∗	–0.180	1.283∗	–2.025∗	–0.495∗	1.560∗	–2.096∗	0.045	1.371∗
	(0.134)	(0.191)	(0.183)	(0.123)	(0.176)	(0.145)	(0.125)	(0.175)	(0.154)
SC2	–0.358∗	0.174‡	0.189‡	–0.329∗	–0.019	0.414∗	–0.304∗	0.017	0.284∗
	(0.068)	(0.098)	(0.107)	(0.062)	(0.090)	(0.103)	(0.063)	(0.089)	(0.074)
SC3	–1.569∗	–0.287‡	1.050∗	–1.500∗	–0.324†	1.119∗	–1.398∗	–0.057	1.004∗
	(0.109)	(0.151)	(0.100)	(0.097)	(0.136)	(0.114)	(0.098)	(0.134)	(0.100)
UA2	–0.763∗	0.088	0.461∗	–0.755∗	0.173†	0.542∗	–0.764∗	0.160†	0.508∗
	(0.066)	(0.089)	(0.085)	(0.057)	(0.080)	(0.081)	(0.061)	(0.082)	(0.061)
UA3	–2.145∗	–0.033	1.193∗	–2.218∗	0.056	1.410∗	–2.007∗	0.342∗	1.297∗
	(0.106)	(0.137)	(0.088)	(0.095)	(0.130)	(0.094)	(0.097)	(0.128)	(0.086)
PD2	–1.244∗	0.016	0.804∗	–1.070∗	0.080	0.845∗	–1.314∗	0.428∗	0.908∗
	(0.071)	(0.095)	(0.073)	(0.061)	(0.085)	(0.073)	(0.068)	(0.091)	(0.069)
PD3	–3.503∗	–0.202	2.000∗	–3.068∗	0.203	2.039∗	–3.743∗	0.795∗	2.156∗
	(0.134)	(0.172)	(0.109)	(0.115)	(0.152)	(0.094)	(0.132)	(0.166)	(0.097)
AD2	–1.288∗	0.141	0.759∗	–0.780∗	–0.016	0.758∗	–0.737∗	0.104	0.649∗
	(0.075)	(0.098)	(0.074)	(0.061)	(0.086)	(0.068)	(0.062)	(0.085)	(0.074)
AD3	–3.760∗	0.031	2.167∗	–2.576∗	–0.122	1.956∗	–2.543∗	0.529∗	1.66∗
	(0.137)	(0.172)	(0.104)	(0.104)	(0.144)	(0.088)	(0.103)	(0.136)	(0.089)
Observations	26 100			26 865			25 605		
Log likelihood	–9691.24			–12 447.34			–11 536.29		
AIC/BIC	19 532.48/20 145.21			25 044.69/25 659.58			23 222.57/23 833.86		
Wald/LR test	32.66∗/32.53∗			42.38∗/32.42∗			60.8∗/45.02∗		

*Note.* Standard errors in parentheses.

2 indicates some problems.

3 indicates a lot of problems.

AD indicates anxiety or depression; AIC, Akaike information criterion; BIC, Bayesian information criterion; LR, likelihood ratio; MO, mobility; PD pain or discomfort, SC, self-care; UA, usual activities.

∗ *P*<.01.

† *P*<.05.

‡ *P*<01.

AD indicates anxiety or depression; PD, pain or discomfort; MO, mobility; SC, self-care; UA, usual activities.

AD indicates anxiety or depression; PD, pain or discomfort; MO, mobility; SC, self-care; UA, usual activities.

The rank order of the dimension levels between adults and adolescents (Fig. 3) differs slightly in all 3 countries. In Germany, only the rank order of AD2 and PD2 was reversed. In Slovenia, the rank order of the MO and UA of the adolescents was reversed compared with the adults (both level 3 and level 2). MO was ranked higher by adolescents, and UA was ranked lower. In Spain, where the adolescents ranked severe problems in mobility higher, rank order was reversed in the MO level 3 and AD level 3 dimensions. On level 2, the rank order was reversed in UA and AD, where adolescents ranked some problems in UA lower.

## Discussion

This is one of the few studies comparing adolescents’ preferences toward EQ-5D-Y-3L health states with those of adults taking the perspective of a child, using DCE valuation tasks and following the international EQ-5D-Y-3L valuation protocol. The use of identical methodology in all 3 countries enables a direct comparison of preferences.

In this study, a DCE was conducted in a sample of adults and in a sample of adolescents in 3 countries: Germany, Slovenia, and Spain. Mixed logit models were used to estimate latent scale utilities for the EQ-5D-Y-3L health states, based on stated preference data obtained from a DCE. The study did not set out to derive value sets. The main result of the analyses indicates that the adolescents’ preferences for EQ-5D-Y-3L health states differ from those of adults valuing health states of a child. Significant differences were also found in a study by Mott et al,^14^ which used a similar methodology in a study in the United Kingdom.

Nevertheless, differences were small and there were almost no statistically significant differences in Germany. Overall, the adolescents gave more importance to MO and SC and less importance to UA, PD, and AD than adults. In particular, the rank order of the dimension levels differed between the adults and adolescents in all 3 countries, with adolescents placing a higher weight on MO level 3 in Slovenia and Spain. Adults may be less concerned with mobility, owing to having different mobility expectations. The frequencies of reported problems with mobility in this study were higher among adults than among adolescents. Therefore, owing to the adaptation phenomenon,^26^ the adults may have seen them as a consequence of aging and were desensitized to them owing to already experiencing them. Furthermore, this result could be due to a lower likelihood of adolescents having mobility issues, resulting in less experience with this dimension and, therefore, an overestimation of its importance in comparison with adults.

Apart from Germany, where significant differences were observed only within the SC dimension, adults in Slovenia and Spain attached higher importance to PD; higher importance being attached to the PD dimension by adults than by adolescents had been found earlier by Mott et al.^14^ Dalziel et al^11^ found that in Australia, adults rated having no PD as best, and in Spain, both adults and adolescents rated no PD as best. Ratcliffe et al^12^ used a best-worst scaling DCE for health states defined by the CHU-9D and found that adults, in general, placed less importance on impairments in mental health (worried, sad, annoyed) and more importance on moderate to severe levels of pain relative to adolescents. As with mobility, the underlying reason could be the experience aspect, because, on average, the adults have gone through more pain and suffering than adolescents.

If younger individuals should be asked to value health states, this brings up the challenge of choosing a preference elicitation technique that can be used in this age group. In previous research, children were shown to struggle with valuation exercises such as time trade-off (TTO),^27^ whereas other studies^11^^,^^14^^,^^28^ showed that children and adolescents performed successfully. Although it is not broadly accepted that children and adolescents aged 11 to 17 years can provide valid responses, there is mounting evidence available that younger individuals are capable of valuing health states using DCE without duration.^10^^,^^14^^,^^29^^,^^30^ In this study, the adolescents completed the DCE tasks quicker than the adults and made fewer inconsistent decisions with the dominant pairs. At a cutoff time of 150 seconds, which was set as one of the quality criteria, more adolescents than adults completed the DCE task without logical inconsistencies. Differences in the validity of responses were not consistent across all 3 countries because adolescents in Germany and Slovenia made fewer logical inconsistencies than adults, and adolescents in Spain made more, regardless of the cutoff time chosen for quality control.

As mentioned earlier, the standard DCE technique produces relative preferences on a latent, or undefined, scale, meaning that this technique does not automatically provide utilities that are anchored to a 0-1 dead-full health scale. Therefore, anchoring is required, which provides an additional post hoc challenge.^6^^,^^27^ To maintain consistency with the tradition of using TTO in the valuation of EQ-5D instruments, the valuation protocol recommends cTTO values obtained via face-to-face interviews to use for anchoring purposes.^5^

One of the limitations of this study was the different perspective used for both samples, which affected the process of preference elicitation^29^, ^30^, ^31^, ^32^, ^33^; it was not possible to determine whether the differences were caused by the perspective of the task or individuals’ preferences. In this study, the adult sample was asked to think about a 10-year-old child during the process of valuation (third person perspective), without specification on who that 10-year-old child was, whereas the adolescents were stating their own preferences. Nevertheless, no 10-year-olds were included in the adolescent samples. The decision to frame the questions for a 10-year-old child followed the published international EQ-5D-Y-3L valuation protocol,^5^ where the framing of the valuation task was “Considering your views about a 10-year-old child, which health state do you prefer?” The cognitive process of imagining who that 10-year old child was may have differed between the respondents and may have affected the preferences.

The given age of a child (10 years) may have also influenced the result of the valuation task, because the preferences of people may differ when considering an 8-year-old child or a 15-year-old adolescent. Craig et al^22^ showed that the values attached to HRQoL losses are different at different imagined ages of a child. The EQ-5D-Y-3L questionnaire was developed for measuring the HRQoL of children and adolescents aged 8 to 15 years, and the specific age of the 10-year-old child was chosen to help respondents to imagine a child within the recommended age range. With this instruction, the age of child that the respondent is thinking about is known to the researcher. Further research is needed to explore the differences in health state values that the adults attach to children and adolescents of different ages within the EQ-5D-Y-3L recommended age range.

The study was conducted as an online survey. Hence, it could not reach a certain population with limited access to the internet or those who do not have sufficient technical skills, such as certain groups of older adults or those living in remote areas. Online survey methods have been linked with concerns as to whether the participants properly engage with and understand the task. Because the researchers had no face-to-face contact with the participants, it was not possible to directly estimate the lack of understanding, engagement, or interest of the participant in the task. To lower the impact of this limitation, we used the quality control procedure explained earlier.

Further research should also be conducted to explore the impact of various variables, such as age, gender, education, perspective, experience, and one’s own health state, on the preferences.

## Conclusions

Our study confirmed the capability of adolescents to complete DCE tasks, confirming that the preferences of adolescents toward EQ-5D-Y-3L are valid. The adolescents’ preferences for EQ-5D-Y-3L health states differed to those of adults considering a child living in the health states valued in all 3 countries. Even though the results confirmed statistically significant differences between severity levels within the dimensions, these differences fade when comparing the importance of relative attributes, especially for Germany. The question remains as to which normative arguments are taken into account in the calculation of EQ-5D-Y-3L value sets. The taxpayer perspective seems reasonable to use in economic evaluations; nevertheless, the perspective of children and adolescents is justifiable as well because their health states are directly affected by selected treatments. Further qualitative research should explore the desirability and acceptance of decision makers and ways of using children’s and adolescents’ preferences.

## Article and Author Information

**Author Contributions:***Concept and design:* Ramos-Goñi

*Acquisition of data:* Prevolnik Rupel, Ramos-Goñi, Ogorevc, Kreimeier, Ludwig, Greiner

*Analysis and interpretation of data:* Ramos-Goñi, Ogorevc

*Drafting of the manuscript:* Prevolnik Rupel, Ogorevc

*Critical revision of the paper for important intellectual content:* Prevolnik Rupel, Ramos-Goñi, Kreimeier, Ludwig, Greiner

*Statistical analysis:* Ramos-Goñi, Ogorevc

*Provision of study materials or patients:* Prevolnik Rupel, Ramos-Goñi, Kreimeier, Ludwig

*Obtainingfunding:* Prevolnik Rupel, Ramos-Goñi, Kreimeier, Ludwig, Greiner

*Administrative, technical, or logisticsupport:* Prevolnik Rupel, Ramos-Goñi, Kreimeier, Ludwig

*Supervision:* Ramos-Goñi, Greiner

**Conflict of Interest Disclosures:** Dr Prevolnik Rupel reported receiving grants from the European Union’s Horizon 2020 research and innovation program and from the EuroQol Group during the conduct of the study. Drs Prevolnik Rupel, Ramos-Goñi, Kreimeier, Ludwig, and Greiner are also members of the EuroQol Group. Drs Ramos-Goñi, Ogorevc, Kreimeier, and Ludwig reported receiving grants from the European Commission during the conduct of the study. Dr Ogorevc reported receiving grants from the EuroQol Research Foundation during the conduct of the study. No other disclosures were reported.

**Funding/Support:** This research was funded by the European Union’s Horizon 2020 research and innovation program and undertaken under the auspices of IMPACT_HTA (grant no. 779312; www.impact-hta.eu) and the EuroQol Group (EQ Project no. 20180510).

**Role of the Funder/Sponsor:** The funders had no role in the design and conduct of the study; collection, management, analysis, and interpretation of the data; preparation, review, or approval of the manuscript; and decision to submit the manuscript for publication.

## Figures and Tables

**Appendix 1:**
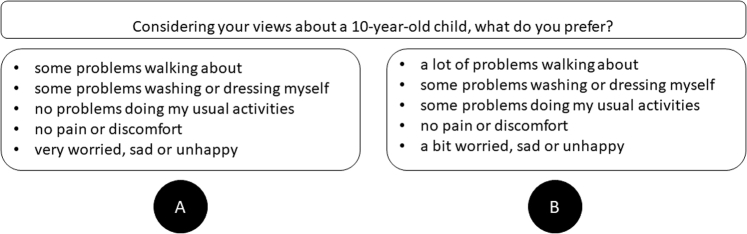
Example of a DCE task in the study (for adults). DCE indicates discrete choice experiment.
